# Medical News and Miscellany

**Published:** 1892-06

**Authors:** 


### Medical News and Miscellany.

See new advertisement of College of Physicians and Surgeons, Chicago, and write for catalogue.

**Dr. R. M. Swearingen**, by invitation delivered an address before the Alamo Literary Society of the Southwestern University at Georgetown on the 6th inst.

**Removals.**—Dr. T. C. Karnes has removed from West Point to Gonzales.

Dr. G. D. Lain has removed from Camp Colorado to Bolivar, Denton county, Texas.

**This Journal.**—It dees not amount to much perhaps, but we beg to announce to whom it may concern that the title of this publication is DANIEL'S TEXAS MEDICAL JOURNAL, and not "Daniel's Medical Journal."

---

**Died.**—Dr. M. L. Crow, an old and highly respected physician of Stephenville, Erath county, Texas, died on 3d of May ult. He had been a member of the State Medical Association for many years and a staunch supporter of this JOURNAL from its |foundation in '85.

---

**Dr. W. N. Rogers**, of Belton, left on June 6th for a tour of northern cities, where he will visit the leading hospitals and attend a course at one of the Polyclinics or P. G. schools. We regret to learn that the Doctor finds this trip necessary, in consequence of impaired health, the result of that curse *la grippe.*

---

**Superfetation Extraordinary.**—A mare belonging to a well-known gentleman in Austin gave birth to a healthy colt about four months ago, and to another last week, both colts are living and doing well. This is authentic; it is vouched for by the owner of the mare and a prominent physician of Austin whose word is authority.

---

**Electrocution a Failure.**—It seems that the costly plant established in Albany, N. Y., for the electrocution of criminals is to be laid away in the attic with other rubbish. The legislature of New York have reported a bill to that effect, hanging to be substituted. We suggest Dr. Hammond's remedy—penitentiary, hard labor and emasculation.

---

**The Rio Chemical Company**, of St. Louis, if it had never done more than present to the profession its valuable Extract of Pinus Canadensis, would have placed the profession under a lasting obligation to it. There is no more healthful, stimulating and generally beneficial application that can be made to a diseased mucous membrane than this.—*Ex.*

---

**Texas students** will find it worth their while to correspond with Prof. F. D. Sim, Dean Memphis Medical College, before matriculating. Their announcement is in this issue. The Memphis Medical College has always been popular with Texans, and in the large class of 1891-2 there were sixty-five Texans. Write for a catalogue. Mention the JOURNAL.

**A Prize of $100 for Best Essay** to "show up the ridiculous pretensions of modern homeopathic practice" will be awarded by Dr. George M. Gould of the *Medical News*, 1004 Walnut street, Philadelphia. The essay to contain not over 15,000 words, and should be adapted in language to the comprehension of lay readers. Send in before January, 1893. For particulars address as above.

---

**The "Old Jeff" Medical College.**—Alumni will be pleased to learn that Jefferson Medical College has purchased two large lots on Broad street, 150x300 feet, and a new and grand building will be erected thereon. It will contain departments for everything and the chemical laboratory and the bacteriological laboratory, while under one roof, will be out of reach of the noise of the tramping of classes going to and from clinics and lectures. The equipment is to be in keeping with the building and fully up to the most advanced methods of instruction.

---

**The Vanderbilt.**—Prof. W. L. Nichol, the able and courteous Dean of the Vanderbilt Medical College, sends the College announcement out as usual in this issue of the JOURNAL. Attention is called to it. Of the many Texas students who go to lectures annually, a very large proportion matriculate at the Vanderbilt. It is a favorite with Texans, and Prof. Nichol is, especially, very popular with the boys. This is another institution which has made a name by pluck, energy and ability. It is one of the famous schools of America. Write to Prof. Nichol for particulars, mentioning the JOURNAL.

---

**An Opening.**—Doctor, if you are seeking a good location' to practice and conduct a drug business, you can hear the particulars of a rare chance by addressing me. Income over $2000 a year; no bad debts; everybody pays up, as it is in the midst of a good cattle and farming section, with educational, social and religious advantages. Only $1200 required. Reason: Owner wants to go to a city. A prosperous town in northwest Texas. Will sell drug business, office fixtures, etc., and can turn over practice and influence. Only one other physician in the county.

Address: Dr. C. M. S., care DANIEL'S TEXAS MEDICAL JOURNAL, Austin.

The Louisville Medical College has its annual announcement in this issue. This college is and has been a popular school with Texans, and Texas students have often taken the prizes there. This school is in a very prosperous condition, and is erecting a new and magnificent building to accommodate the growing classes. The new college building now in process of construction is to cost $150,000, and will be completed in time for the fall term. The *Courier-Journal* says of this enterprising Faculty:

"Without any indorsement, without any outside assistance, with nothing but a reputation for careful teaching, and a large, enthusiastic and faithful corps of alumni to sustain them, the Louisville Medical College has entered upon this stupendous work. It is regarded by them as a proud monument to faithful and conscientious work."

Write for a catalogue to Dr. Geo. M. Warner, the courteous Secretary, and mention this journal.

---

Acne.—In the clinic, for a case of acne vulgaris in a young woman aged nineteen years, Dr. Henry W. Stellwagon gave the following treatment: At night wash the face with soap and water, and then steam the face or wash with as hot water as can be borne. Afier doing this make a thorough application of the following lotion so that the sediment will be well spread over the face:'

| | | |
|---|---|---|
| ℞ | Zinci sulphat | ʒj |
| | Potassii sulphurat | ʒj |
| | Aquæ | ℥ij |
| | Alcoholis q. s | ℥iv |

M.

Dissolve the salts separately, each in ʒj of the water aud then mix with the alcohol. The bottle should be well shaken before the lotion is applied, as the sediment is the part that does the most good.

The patient's bowels should be kept freely open and all secretions active. Also give internally sulphide of calcium in doses of 1-10 of a grain in gelatine-coated pills three times a day.—*Medical Review.*

---

Dr. L. Y. Grubbs, of Topeka, (*Kansas Medical Journal*), was called by the court to make an examination of a case in a criminal case and testify in the case. The doctor asked the court to excuse him because of urgent professional engagements. The court refused the request. The doctor demanded an expert fee, saying that his time and professional knowledge was his capital. The court refused to comply with his demand. Dr. Grubbs then said: "I have never examined or seen the case, and know nothing about it, and I now demand an expert fee in advance for rendering thiss ervice—for obtaining information for the court which requires my time and professional knowledge." The court then ruled that the doctor could not be compelled to render such service without being paid in advance on his demand. Dr. Grubbs' example deserves imitation, and some of the money now wasted by medical societies in sustaining "organs," could be profitably expended in carrying such cases to courts of supreme appeal.

